# Randomised controlled trial comparing single agent paclitaxel *vs* epidoxorubicin plus paclitaxel in patients with advanced ovarian cancer in early progression after platinum-based chemotherapy: an Italian Collaborative Study from the ‘Mario Negri’ Institute, Milan, G.O.N.O. (Gruppo Oncologico Nord Ovest) group and I.O.R. (Istituto Oncologico Romagnolo) group

**DOI:** 10.1038/sj.bjc.6601787

**Published:** 2004-04-20

**Authors:** A Buda, I Floriani, R Rossi, N Colombo, V Torri, P F Conte, R Fossati, A Ravaioli, C Mangioni

**Affiliations:** 1 Istituto di Ricerche Farmacologiche ‘Mario Negri’, Milan, Italy; 2 Ospedale San Gerardo, Monza, Italy; 3 European Institute of Oncology, Milan, Italy; 4 Ospedale S Chiara, Policlinico Universitario, Pisa, Italy; 5 Ospedale degli Infermi, Rimini, Italy

**Keywords:** randomised trial, early progression, chemotherapy, recurrent ovarian cancer

## Abstract

The aim of the study was to evaluate the role of epidoxorubicin plus paclitaxel combination (ET) *vs* single agent paclitaxel (T), as second-line chemotherapy treatment in advanced ovarian cancer patients in early progression within 12 months after platinum-based chemotherapy. From October 1994 up to June 1999, 234 patients from 34 Italian hospitals were randomised to receive: (A) epidoxorubicin (E) 80 mg m^−2^ + paclitaxel (T) 175 mg m^−2^ (3 h infusion), every 21 days for 4–6 cycles. (B) Paclitaxel 175 mg m^−2^ (3 h infusion) every 21 days for 4–6 cycles. Evaluable for survival analysis were 106 and 106 patients in ET and T arm, respectively. Platinum-based monochemotherapy was the first-line treatment in 43% patients, while polichemotherapy containing anthracyclines was the preferred first-line therapy in 22% patients. The median time from the end of first-line therapy to randomisation was 3 months. Treatment was completed in 87 and 85% of T and ET arm, respectively. Haematological toxicity was significantly more common in ET group (ECOG grade 3–4 neutropenia: 37.4% in ET *vs* 18.2% in T arm). Neuropathies were similar in both arms (sensory: ECOG grade 2–3: 12.1% in ET *vs* 14.7% in T arm, motor: 6.1% in ET *vs* 5.3% in T arm). Objective response was achieved in 37.4% of patients in ET group and in 46.9% of patients in T arm. At a median follow-up of time of 48 months, a total of 180 patients progressed and 163 patients died. Survival analysis showed no difference between ET and T (median time to progression: 6 months for both regimens, median survival: 12 and 14 months for ET and T, respectively; hazard ratio for mortality of ET *vs* T: 1.17 (95% CI 0.86–1.59; *P*=0.33). The ET regimen does not seem to be more effective than T in refractory advanced ovarian cancer patients in early progression after platinum-based chemotherapy. Despite an acceptable response rate, the control of disease progression remains poor.

Ovarian cancer, fifth cause of cancer death in European women, is the most common cause of death among women with gynaecological malignancies. An estimated 25 200 new cases of ovarian cancer are diagnosed annually in the US and about 14 500 women die every year (Landis *et al*, 1999). Ovarian cancer is often diagnosed in advanced phase (stage III and IV) and the prognosis is generally poor despite activity shown by chemotherapy agents. Although up to 80% of cases achieve an objective response with platinum-containing regimens, about 22% of patients progress while receiving initial platinum-based treatment and are defined refractory to chemotherapy (Kavanagh *et al*, 1995), 40% of patients generally relapse or progress within 12 months and 20% after 12 months from the end of first-line chemotherapy (Ozols and Young, 1991; Thigpen, 1999). The probability of a second response increases with the relapse-free period (Seltzer *et al*, 1985; Gore *et al*, 1990; Markman *et al*, 1991; Weiss *et al*, 1991; Zanaboni *et al*, 1991; Eisenhauer *et al*, 1997; Cantù *et al*, 2002).

For these patients, second-line treatment include drugs that are noncrossresistant with platinum compounds including ifosfamide, anthracyclines at standard or high dose (Markman *et al*, 1992; Thigpen *et al*, 1993; Vermorken *et al*, 1994; Vermorken and Pecorelli, 1996) and, more recently, taxanes.

In a review analysis, Vermorken *et al* (1999) showed that 25 out of 75 (33%) chemotherapy patients from several trials achieved an objective response to single agent doxorubicin. The results of single agent epirubicin appear to be better in prior platinum-exposed patients. Paclitaxel, an antimitotic agent derived from bark of Pacific Yew tree *Taxus brevifolia* (Schiff *et al*, 1979), proved to be active in relapsed and platinum-resistant ovarian cancer (McGuire *et al*, 1989; Einzig *et al*, 1992; Trimble *et al*, 1993; Kohn *et al*, 1994; Gore *et al*, 1995) and was well tolerated by most patients: significant side effects other than alopecia were uncommon (Seidman *et al*, 1996). Moreover, preliminary data in advanced breast cancer patients seemed to suggest that the combination of paclitaxel plus doxorubicin might have a potential interaction in efficacy (Gianni *et al*, 1994). In absence of sound data to support the use of paclitaxel as single agent or in combination with anthracyclines in the second-line treatment of ovarian cancer, we set up a randomised multicenter phase III trial. This trial aimed at evaluating the efficacy and feasibility of paclitaxel as single agent compared to a combination including paclitaxel and epidoxorubicin, administered as second-line chemotherapy for epithelial ovarian cancer patients progressing within 1 year from the end of first-line platinum-based chemotherapy. Patients exposed to largely sub-maximally cumulative doses of anthracyclines in first-line platinum-based chemotherapies were deemed still eligible for this study.

## MATERIALS AND METHODS

### Design

The study was run as parallel trial, adopting a randomisation ratio between arms of 1 : 1. Randomisation was performed calling the coordinating centre located at the Istituto di Ricerche Farmacologiche ‘Mario Negri’, Milan. A stratification was adopted considering centre and response achieved after first-line chemotherapy, in terms of progression during treatment (refractory patients), partial or stable disease, relapse within 12 months.

### Ethics committee

The study protocol was revised and accepted by local ethical committees; informed consent was obtained before randomisation from all patients in accordance with national legislation following the principles enunciated in the Declaration of Helsinki (Vastag, 2000).

### Eligibility

Patients with histological proven ovarian carcinoma platinum resistant, relapsed or progressed within 12 months since the end of a first-line therapy containing cisplatin or carboplatin, were eligible for the study. Further eligibility criteria included: measurable disease, World Health Organization (WHO) performance status ⩽2; adequate bone marrow function (absolute granulocyte count ⩾2 000 mm^−3^, platelet count ⩾100 000 mm^−3^); adequate renal, hepatic and cardiac functions. Patients were considered not eligible if they had received more than one previous chemotherapy line or if the first-line chemotherapy contained taxanes. Prior anticancer treatment with anthracyclines was allowed provided patients had not received cumulative doses in excess of 300 or 360 mg m^−2^ for doxorubicin or epidoxorubicin, respectively.

### Chemotherapy regimen

Patients were randomised to receive T or ET, intravenously, at 3-week intervals, as soon as possible after randomisation.

Paclitaxel was given 175 mg m^−2^ over 3 h infusion. Premedication given to reduce the risk of paclitaxel hypersensitivity, was as follows: Desamethasone 40 mg, given as 20 mg at 12 and 6 h before paclitaxel, 300 mg of Cimetidine and 10 mg of Chlorpheniramine intravenously, 30 min before the treatment. Epidoxorubicin 80 mg m^−2^ intravenously was administered as a bolus followed by paclitaxel 175 mg m^−2^ over 3 h infusion, as already described. Each patient had to receive a minimum of four cycles of chemotherapy but patients with progressing disease at the first 2 month evaluation were considered off study and the assigned treatment was interrupted. After completion of four cycles, two additional cycles of protocol treatment were proposed to all patients not showing disease progression. Patients were allowed to receive any third-line chemotherapy at investigators' discretion.

### Treatment modifications

Toxiciy was graded on a scale of 1–4 according to the WHO criteria. If, at the time of scheduled retreatment, white blood cell count was ⩾1 500 mm^−3^ and platelets count ⩾100 000 mm^−3^, chemotherapy was given without reductions or delays, otherwise it was delayed by 1 week or reduced according to type and grade of toxicity. The following indications of toxicity led to dose modification: (a) nadir neutrophil count between 500 and 1 000 mm^−3^ or nadir platelet count between 50 000 and 100 000 mm^−3^: dose level – 1 (see below); (b) nadir neutrophil count less than 500 mm^−3^ or nadir platelet count less than 50 000 mm^−3^: dose level – 2 (see below); (c) any nonhaematologic grade 2 toxicity: dose level – 1; (d) any nonhaematologic toxicity more than grade 2: treatment to be interrupted until the adverse effects resolve. Dose levels were as follows for paclitaxel and epidoxorubicin, respectively. Initial dose, 175 and 80 mg m^−2^; level – 1, 135 and 65 mg m^−2^ ; level – 2 110 and 50 mg m^−2^.

### Assessment of response

Response to study drug, assessed using ECOG criteria (Miller *et al*, 1981), was based on objective tumour evaluation every two cycles.

A complete response (CR) was defined as complete disappearance of all measurable and assessable lesions no new lesions and no related symptoms. A partial response (PR) was documented in patients with a ⩾50% decrease in the sum of the products of bidimensional perpendicular diameters of all measurable lesions. Progressive disease (PD) was said to occur in patients with a ⩾50% increase in the sum of the products of bidimensionally measured lesions over the smallest sum obtained at best response, or clear worsening of any assessable disease, or failure to return to evaluation because of death or deteriorating condition, or the appearance of any new lesion or site. Patients were classified as having stable disease if they did not qualify for CR, PR or PD.

### Follow-up programme

Once patients were off the protocol treatments, they were monitored for assessment of disease status and long-term toxicities every 3 months for 2 years and every 6 months thereafter. Follow-up procedures comprised clinical examination, blood chemistry and CA 125 (optional) estimation. ECG and echocardiography for cardiac function assessment were scheduled at 6 and 12 months and yearly thereafter; routine computed tomography, abdomen echography or chest radiography were not required but were requested if biomarkers rose or symptoms developed.

### Analysis

The primary outcome measure was overall survival; secondary outcomes were progression-free survival and response to treatment.

The trial was designed to have an 80% power to detect a 33% relative reduction in mortality (i.e. increasing median overall survival of about 6 months), corresponding to a hazard ratio of 0.67 with a two-sided *α* of 0.05. We anticipated that 230 patients would have to be recruited to the trial to meet these specifications. The final analysis was designated to occur when at least 85% of the eligible patients experienced either progression or death.

We compared Kaplan–Meier curves for overall survival and progression-free survival using the Mantel–Cox version of the log-rank test (Kaplan and Meier, 1958; Makuch and Simon, 1982). The Cox proportional hazards regression model (Parmar and Machin, 1995) was also used for progression-free and overall survival to estimate the treatment relative hazards (HRs), while adjusting for multiple prognostic factors.

Response to chemotherapy treatments was compared with Mantel–Haenszel *χ*^2^ test for trend.

Overall survival was defined as the time from randomisation to death from any cause; patients known to be still alive at the time of the analysis were censored at the time of their last follow-up. Progression-free survival was defined as the time from randomisation to first appearance of PD or death from any cause; patients known to be alive and without PD at the time of analysis were censored at the time of their last follow-up. Absolute benefits at specific time points were calculated using the Kaplan–Meier estimate of survival in the control group at the time point (control survival) and hazard ratio with the expression: absolute benefit=exp (hazard ratio × log (control survival))−control survival. This approach was also adopted for the end point of progression-free survival. Although this approach implicitly assumes proportional hazards, it is preferable to reading differences between Kaplan–Meier curves at individual time points. Differences in medians were calculated in a similar way but using the expression difference in medians=(control median/hazard ratio)−control median. This approach assumes approximately exponentially distributed survival times. The relative benefits of platinum-based chemotherapy on survival were assessed in an exploratory manner in subgroups defined by prior use of anthracyclines and refractory status. To test for differences in the relative size of effect in different subgroups, we used a *χ*^2^ test for interaction, or, when appropriate, a *χ*^2^ test for trend.

All analyses were performed on an intention-to-treat basis on eligible patients except for the analyses of toxicity. The latter analyses were restricted to all patients who received at least one cycle of allocated treatment. All *P*-values are two-sided. Analyses were carried out using SAS System Version 8.20. No interim analysis was planned or performed.

## RESULTS

### Accrual

**Figure 1:**
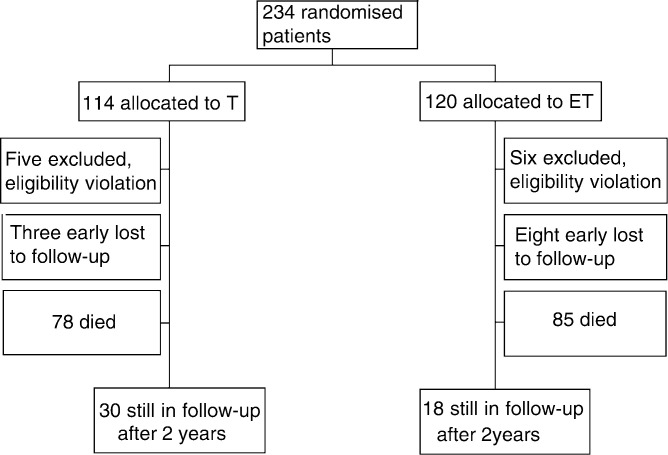
Flow chart of the progress of patients through the trial

Between October 1994 and June 1999, 234 patients were enrolled into the trial from 34 Italian centres. Final survival analysis was performed on 212 patients, as there were 11 major violations of the protocol (six patients were given paclitaxel in first-line therapy, two had more then one first-line therapy, two patients had a disease-free interval greater than 12 months and one patient had cardiac impairment) and 11 further patients were early lost to follow-up. Figure 1

reports the flow chart of the progress of patients through the trial.

### Patient characteristics

Pretreatment characteristics were well balanced within randomised groups as listed in Table 1

**Table 1:** Patient characteristics

	**T**	**ET**
Evaluable patients	**106**	**106**
Age – Median (min–max)	59 (24–78)	60 (28–77)
*Performance status*		
0–1	99 (93.4%)	97 (91.5%)
2–3	7 (6.6%)	9 (8.5%)
*Previous treatment*		
Monochemotherapy	48 (45.3%)	43 (40.6%)
Polichemotherapy no anthracyclines	29 (27.4%)	31 (29.2%)
Polichemotherapy with anthracyclines	23 (21.7%)	24 (22.6%)
Unknown	6 (5.7%)	8 (7.5%)
Months from the end of first-line chemotherapy		
Median (min–max)	3 (0–12)	3 (0–12)
*Time of occurrence of progression*		
During first line chemotherapy	33 (31.1%)	39 (36.8%)
Within 6 months from the end of first-line chemotherapy	46 (43.4%)	37 (34.9%)
6–12 months from the end of first-line chemotherapy	26 (24.5%)	29 (27.4%)
Unknown	1 (0.9%)	1 (0.9%)

T=paclitaxel; ET= epidoxorubicin plus paclitaxel. Bold values indicate the number of patients (*n*).

: median age was about 60 years, platinum-based monochemotherapy was the first-line treatment in 43% of patients. Polichemotherapy containing anthracyclines was the preferred first-line therapy in 22% of patients. Progression during first-line chemotherapy was observed in 32% of cases. Median time from the end of first-line chemotherapy was 3 months.

### Compliance and toxicity

In all, 87 and 85% of the patients completed the scheduled therapy in T and ET, respectively. However, modification of dosage or time accounted for 9 and 41% of cases (*P*<0.001), and interruption due to toxicity were 9 and 13% (*P*=NS), respectively. Four percent of patients in both arms refused therapy after randomisation. A median number of six courses, with an interquartile range from 4 to 6, was given to T group and a median number of five courses, with an interquartile range from 3 to 6, was given to ET group. Table 2

**Table 2:** Toxicity evaluation

	**T**	**ET**	***P***
Evaluable patients	**99**	**99**	
*Haematological grade III–IV*			
Leucopenia	9 (9.1%)	19 (19.2%)	0.06
Neutropenia	18 (18.2%)	37 (37.4%)	0.01
PLT	1 (1.0%)	1 (1.0%)	0.58
HB	5 (5.0%)	3 (3.0%)	0.10
*Non haematological*			
Neuropathy (II–III)			
- Sensorial	14 (14.1%)	12 (12.1%)	0.32
- Motor	5 (5.0%)	6 (6.0%)	0.83
Cardiac (II–III)	2 (2.0%)	1 (1.0%)	0.41
Nausea, vomiting (III–IV)	6 (6.0%)	11 (11.1%)	0.36
Alopecia (III–IV)	68 (68.7%)	61 (61.6%)	0.24

T = paclitaxel; ET = epidoxorubicin plus paclitaxel. Bold values indicate the number of patients (*n*).

shows the proportion of patients with toxic effects observed during treatment. Levels of haematological toxicity were quite different across the arms. The ET was associated with higher rates of grade 3 or 4 haematological toxicity: leuckopenia and neutropenia. Other nonhaematological toxicities occurred as expected and the toxicity profiles of the two groups were similar.

### Response

Response evaluation results are shown in Table 3

**Table 3:** Response evaluation

	**T**	**ET**
Evaluable patients	**90**	**91**
CR	18 (20.2%)	14 (15.4%)
PR	24 (26.7%)	20 (22.0%)
SD	23 (25.6%)	24 (26.4%)
PD	25 (27.8%)	33 (36.3%)
Median duration of response (mos) (min–max)	8 (1–49)	11 (1–53)

χ^2^ test for trend : 1.89, *P*=0.17. T = paclitaxel; ET = epidoxorubicin plus paclitaxel; CR = complete response; PR = partial response; SD = stable disease; PD = progressive disease. Bold values indicate the number of patients (*n*).

. Data were available in 181 patients. The overall response rate was 46.9% in T (CR: 20.2%; PR: 26.7%) and 37.4% in ET (CR: 15.4%; PR: 22.0%), while PD was observed in 27.8 and 36.3% of patients, respectively (Mantel–Haenszel *χ*^2^ test for trend: 1.89, *P*=0.17). The overall high rate of overall response was largely due to the patients progressing after 6 months since the end of first-line chemotherapy. In fact, a subset analysis in this population showed an overall response rate as high as 60% in T and 48% in ET.

### Overall survival

**Figure 2:**
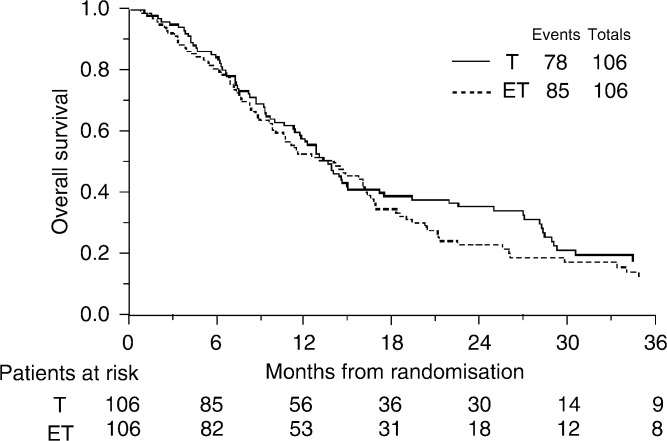
Overall Survival

At a median follow-up of 48 months, 163 (76.9%) patients have died. Comparing the survival curves (Figure 2

), the hazard ratio of 1.17 (95% CI: 0.86–1.59; *P*=0.33) translates into an absolute difference in 1-year survival of 6% (95% CI for difference: −5 to 16%) in favour of single agent paclitaxel, from 50 to 56%. In terms of median survival, the hazard ratio translates into a difference of 2 months (95% CI for difference: -2 to 5 months) with a median survival of 14 months on the paclitaxel alone arm and 12 months on the paclitaxel plus epidoxorubicin chemotherapy arm.

### Progression-free survival

**Figure 3:**
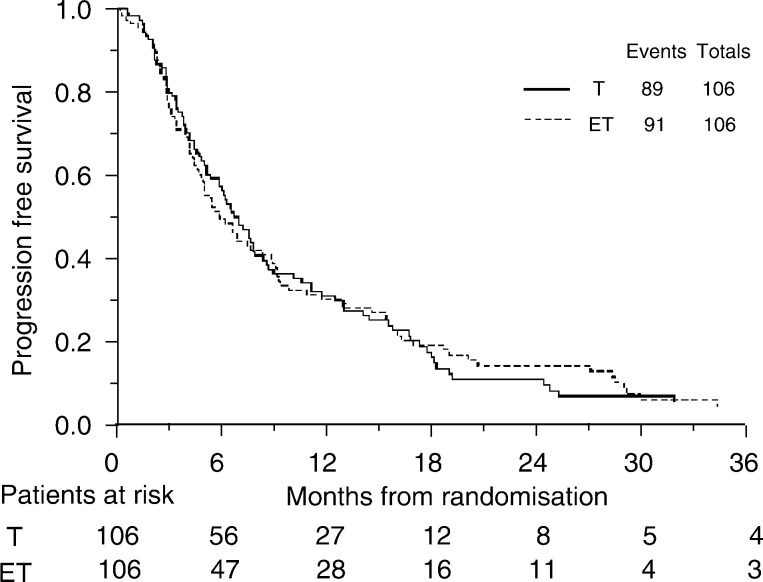
Progression-free survival

A total of 180 (84.9%) patients have progressed or died. Progression-free interval curves are shown in Figure 3

. The hazard ratio of 1.00 (95% CI: 0.75–1.35; *P*=0.96) translates into an absolute difference in 6-months survival of 0% (95% CI for difference: −10 to 10%). In terms of median survival, the hazard ratio translates into a difference of 0 months (95% CI for difference: −2 to 2 months) with a median survival of 6 months on both chemotherapy arms.

### Effects in different subgroups

We found no definite evidence that paclitaxel plus epidoxorubicin was more or less effective than paclitaxel alone in any subgroup for either overall survival or progression-free survival (Table 4

**Table 4:** Effect of chemotherapy in different subgroups

	**PFS**		**OS**	
**Effect of ET *vs* T**	**HR**	**95% CI**	**HR**	**95% CI**
Stratification factors				
*Previous use of anthracyclines*				
No	1.05	0.75–1.46	1.38	0.97–1.96
Yes	0.90	0.48–1.68	0.68	0.34–1.34
*χ*^2^ test for interaction	*P*=0.857		*P*=0.060	
				
*Refractory status*				
No	1.03	0.73–1.47	1.08	0.74–1.58
Yes	0.92	0.54–1.56	1.38	0.80–2.37
*χ*^2^ test for interaction	*P*=0.718		*P*=0.403	

Treatment relative hazards (HRs) HR s more than 1 are in favour of T, HRs less than 1 are in favour of ET. PFS= progression-free survival; OS= overall survival; HR= treatment relative hazards; 95% CI= 95% confidence interval; ET= epidoxorubin plus paclitaxel; T= paclitaxel.

). However, a statistically borderline test for interaction suggests that previous exposure to anthracyclines-based first-line chemotherapy might interact in the relative performance of the two regimens, favouring ET combination.

## DISCUSSION

Salvage therapies in relapsed epithelial ovarian cancer are rarely curative and usually result in low response rates with small impact on survival. The current management of patients with recurrent ovarian carcinoma is based on the results of the initial chemotherapy. In the early 1990s, paclitaxel became the major second-line treatment for ovarian cancer and soon after gained the role of standard first-line treatment in combination with platinum compounds (Thigpen *et al*, 1994; Kavanagh *et al*, 1995). More recently, the negative results of two large-scale clinical trials (Muggia *et al*, 2000; ICON3 group, 2002) have challenged the general consensus on paclitaxel as a first-line therapeutic approach. In accordance with these new evidences, for example, the UK National Institute for Clinical Excellence (NICE) has revised its guidance on the use of paclitaxel in the treatment of ovarian cancer in January 2003 (NICE, 2003) and recommended that paclitaxel in combination with platinum-based compound or platinum-based therapy alone are offered as alternatives for first-line chemotherapy. This means that the patients who were eligible for our study still possibly represent a significant subset of the ovarian cancer patients currently referring to oncologic outpatient's departments. The most important aspect emerging from this study, which, to our knowledge, is one of the largest phase III randomised trials ever performed in recurrent ovarian cancer, is that the combination of two noncrossresistant drugs failed to achieve better results and was associated to an higher toxicity than the single agent paclitaxel in the management of advanced ovarian cancer patients in early progression after platinum-based chemotherapy. Our data can be compared with the results emerged from the study of Bolis *et al* (1999). In this multicenter phase III randomized trial, combination of paclitaxel plus epidoxorubicin gave a higher response rate than single agent paclitaxel. In 81 evaluable patients, the overall response rates was 17% in paclitaxel arm and 34% in the combination arm but, in keeping with our findings, toxicity was higher in combination therapy, duration of response was limited to a few months, and the 2-year survival was similar between the two groups. Typically, response rates to second-line chemotherapy in platinum-resistant recurrent ovarian cancer is modest although overall response rates up to 40% have been reported in the literature (Salom *et al*, 2002). The high proportion of patients achieving an objective response in our study is mainly due to the fact than about a quarter of our study population had a disease-free interval greater than 6 months (but less than 12 months), while most of the series of platinum-resistant patients included only patients recurring within 6 months. However, comparing response rates across studies makes clear that a strong relationship between response and survival is not to be expected in this setting and therefore clinical outcome measures related to survival or quality of life should be preferred for efficacy assessment.

The findings of this trial indicate that the combination of paclitaxel plus epidoxorubicin does not seem to be more effective than single agent paclitaxel in patients in early progression after first-line platinum-based treatment. In subset analyses, the appearance of a statistically borderline test for interaction suggesting a different performance on survival of ET when compared with T in patients who were given anthracyclines in first-line therapy came unexpected. We deemed this finding devoid of a real clinical value apart from confirming that anthracyclines resistance did not develop in these patients.

If the combination of common cytotoxic drugs does not seem to improve survival in patients with refractory ovarian cancer, further studies are necessary to address the open questions regarding the best second-line therapy in the recurrent disease setting. There is now evidence suggesting that drug resistance is partly a result of defects in the apoptotic pathway that, after previous chemotherapy, can determine selection of tumour cells, more resistant to subsequent agents (Cannistra, 2002). Also, the role of paclitaxel has to be further defined: recent data suggest that a possible role of paclitaxel may be in cisplatin-resistant clones of cells that overexpress mutated p53 protein (Smith-Sorensen *et al*, 1998; Judson *et al*, 1999). Large comparative trials of second-line treatment should be planned to test the available new active agents, which have different mechanisms of action and potentially limited serious toxicity, while offering the best framework to improve our knowledge of predictive factors.

## COLLABORATORS AND AFFILIATIONS

Avellino, Casa di Cura Malzoni: A Vernaglia Lombardi, M Malzoni; Centro di Riferimento Oncologico IRCCS, Aviano: S Tumolo; Ospedale Policlinico Universitario, Bari: G Cormio; Ospedale degli Infermi ASL 12, Biella: A Monaco; Ospedale Regionale, Bolzano: F Welponer; Ospedale Cannizzaro, Catania: R Ruggeri, P Scollo; Ospedale L Currò, Catania: D Priolo; Ospedale Generale Valduce, Como: L Redaelli; Ospedale di Circolo, Desio: G Orfanotti; Azienda Ospedaliera S Anna, Ferrara: R Martinello; Ospedale S Antonio Abate, Gallarate: M Borsani, S Garsia; Ospedale Santa Maria Goretti, Latina: M D'Aprile; Azienda Ospedaliera, Lecco: N Natale; Azienda Ospedaliera C Poma, Mantova: G Cavazzini; Istituto Europeo di Oncologia, Milano: G Parma, L Bocciolone; Ospedale San Carlo Borromeo, Milano: MC Locatelli; Ospedale San Gerardo dei Tintori, Università Milano-Bicocca, Monza: MG Cantù, S Chiari; Ospedale Civile, Padova: MO Nicoletto; Università di Padova, Pat. Ginecologica: UM Fiorentino; Ospedale V Cervello, Palermo: D Gueli Alletti, C Strazzeri; Ospedale Civile Agnelli, Savigliano, Cuneo: L Galletto; Unità Sanitaria Locale Roma no 26, Tivoli, Roma: G Corrado; Università Dipartimento Scienze Ginecologiche e Ostetriche, Torino: A Durando; Ospedale Civile Consortile, Treviglio: R Grassi; Azienda Ospedaliera, Verona: C Griso.
